# An Extract from the Report on Diet for the Committee of Research of the American Academy of Periodontology

**Published:** 1923-02

**Authors:** Andrew J. McDonagh

**Affiliations:** Past President, Toronto, Canada


### AN EXTRACT FROM THE REPORT ON DIET FOR THE COMMITTEE OF RESEARCH OF THE AMERICAN ACADEMY OF PERIODONTOLOGY

BY ANDREW J. MCDONAGH, L. D. S., PAST PRESIDENT,

TORONTO, CANADA

The results of Research work which have been carried on in various parts of the world during the last ten years show that conditions of the body are either controlled or greatly influenced by the food partaken of by the individual. This is true of all parts of the body, the periodontal tissues not excepted. Furthermore, during the formative period at least, not only the character of the periodontal but the dental tissues is governed by the food ingested.

The work of Dr. and Mrs. Mellanby seems to leave no doubt as to the foregoing. The experiments of Mrs. Mellanby on dogs go to show that the form and quality of the bones of the jaw and the alignment of the teeth depends to an almost incredible degree on the constituents of the food consumed during the formative period; consequently the early feeding of the growing animal carries its influence so far as the periodontium is concerned, right through the span of life. But not only is food an important factor in the growing animal, the adult as well is affected by the character and constituents of his food. Scurvy which is solely the result of a deficient diet manifests itself as a destroyer of the periodontal tissues, resulting in many cases in one form of the disease we recognize as dental periclasia. Until recently the origin of this phase of dental periclasia was not recognized because we had not thought of scurvy in any sense except as we see it in an extreme case. We are indebted to Dr. Hess, who has taught us that one of the effects of a scorbutic condition, is to cause the blood vessel walls to deteriorate so that the blood will break through and we have a bleeding of the gums before other symptoms of scurvy are manifest. A prolongation or recurrence of this stage of scurvy may be a more frequent factor in the etiology of dental periclasia than is generally conceded.

Dr. Eugene Talbot in his work on "Interstitial Gingivitis" (another name for dental periclasia) gives full credit to the assimilation of food and to metabolism. His work if followed to a logical conclusion will not be far off from the facts deduced from the later study of conditions resulting from a scorbutic diet. But it was left to Dr. Black to prove by his experiments that the amount of food ingested by the individual was responsible for the amount and kind of salivary calculus deposited on the teeth of the individual. By repeated experiments he has shown that if a person takes and can digest so much food that the quantity of globulin and caglobulin manufactured is in excess of the amount the tissue can immediately use or store, these excessive nutritive materials will appear in the fluid secretions of the body, consequently the saliva will be overloaded with them and as a result we have a deposit of salivory calculus lodging on the teeth. The question of vitamines and the balance of the lime salt content of the food undoubtedly will have some influence in this connection, but experiments along this line are not sufficiently conclusive as yet to make a definite statement.

The study of the influence which the endocrine glands exert over dental periclasia is just in its infancy, but apparently the action of these glands is influenced greatly by the constituents of the food. As an instance, the effect of vegetables containing iodine on the Thyroid gland. No doubt the para thyroid and other ductless glands are similarly effected by various food ingredients and we now treat some phases of periodontoclasia with these facts in view.

A year or so ago the members of the Academy of Periodontology received questionnaires which I sent them, also a request that they fill them out and return them to me. Some have complied, a few have not. These questionnaires I also sent to a number of dentists who are not members of the Academy but who might be interested. In all I have had returned about five hundred. Many interesting facts are disclosed by the answers given. The information, however, is more negative than positive. In other words, it tells us that certain food habits which some enthusiasts claim are the cause of dental and periodontal trouble can not be relied on to prove these theories. The diet ordinarily used on this continent by the adult seems to be adequate for nutrition. The answers, however, show that there is a possibility of injury to the periodontal tissues and probably to the dental tissues of the adults, as the following shows.

SUMMARY OF RETURNS

*A good proportion of carbohydrates but lack of raw vegetables and raw fruits.*

26% have healthy periodontal tissues and no pockets.
74% have pockets or unhealthy periodontal tissues.
45% have numerous cavities or fillings.
55% have very few cavities or fillings.

*A good proportion of raw vegetables and raw fruit and lack of carbohydrates.*

24% have healthy periodontal tissues and no pockets.
76% have pockets or unhealthy periodontal tissues.
42% have numerous cavities or fillings.
58% have few cavities or fillings.

*A good proportion of carbohydrates, raw fruits and vegetables.*

36% have healthy periodontal tissues and no pockets.
64% have pockets or unhealthy periodontal tissues.
52% have numerous cavities or fillings.
48% have few cavities or fillings.

*A good proportion of carbohydrates with milk but no raw vegetables or fruit.*

37% have healthy periodontal tissues and no pockets.
62% have pockets or unhealthy periodontal tissues.
56% have numerous cavities or fillings.
44% have few cavities or fillings.

*A good proportion of raw vegetables and raw fruit with milk, but lack of carbohydrates.*

26% have healthy periodontal tissues and no pockets.
73% have pockets with unhealthy periodontal tissues.
36% have numerous cavities or fillings.
64% have few cavities or fillings.

*A good proportion of raw vegetables and fruit, milk and carbohydrates.*

35% have healthy periodontal tissues and no pockets.
65% have pockets with unhealthy periodontal tissues.
58% have numerous cavities or fillings.
42% have few cavities or fillings.

As far as these returns are concerned we must remember that the dentists were asked to send reports of the first cases coming into their office after receiving the questionnaires. Therefore the majority of these are not selected cases and would represent the usual run of patients, in other words the average individual and the average diet, which on the whole is probably fairly well balanced; but a few cases were reported which were exceptional and which I will relate as they bear on certain phases of the case.

One outstanding fact seemed to be evident throughout —the almost universal connection of worry with dental periclasia. Of all the reports received there were less than two per cent. who were reported as not worrying, who had periodontal destruction in any advanced degree. Furthermore, those comprising the two per cent. follow avocations which would ordinarily cause a nervous strain, as for instance school teaching and law. One dentist reported that two patients of his had stopped the habit of worrying after he had treated the cases successfully, another reported the same thing about one of his cases. This would lead one to suspect the nerves as having some connection with dental periclasia.

One of the most interesting reports came from Dr. Fleming, who says a family living in his neighborhood acquired at the same time two dogs of the same age, one a mongrel, the other a pet house dog. The mongrel was put out of the house to live and fed the same as that sort of dog usually is, being allowed to forage part for himself. The other, the house dog was tenderly cared for and fed the same thoroughly cooked food as that which was served the family. The mongrel kept his teeth to old age, in good condition. The house dog lost practically all of his teeth, because they became loose and the investing tissues were destroyed. Was this due to the breeding, the environment, the kind of food, the quantity of food or what?

We all have many cases in our own practices which tell a lesson but we fail sometimes to get all out of it we might. I will close this by relating one which I had to deal with. A young man came to me whose gingival tissues surrounding the upper anterior teeth, from cuspid to cuspid were enlarged to twice their normal size, extending over the teeth to near the incisal edge, composed apparently of fairly dense fibrous tissues of a very dark color. There was a scant exudation from the gingival crevices which were of course unusually deep. This exudation ceased, however, when the gingival crevices were thoroughly cleaned out. There was scarcely any bleeding even under pressure or manipulation or instrumentation. The color which I spoke of was very dark blue in some places almost black, as this picture which I am showing you will bear out. This picture by the way was made by Dr. Milne from the case while the patient sat in the chair and is a good representation of the case. This patient was a fruit grower and fruit salesman and practically lived on apples, eating as many as two dozen large ones at a time, without taking any other kind of food. By changing his diet and by local treatment we were able to reduce the size of his anterior ula to nearly normal and to induce a better color. Was this due to endocrine disturbance or to fruit acid effecting the lime salts of the blood, or some other cause?

This report which is really one of progress is I know very disappointing in as much as there is so little of a conclusive nature in it but in conjunction with other work of this kind it may be of some use. I sincerely hope it will or rather I sincerely hope some one will take up this work and do what, up to the present I have failed to accomplish.

